# Investigation of Electrostatic Effects on Enyzme Catalysis: Insights from Computational Simulations of Monoamine Oxidase A Pathological Variants Leading to the Brunner Syndrome

**DOI:** 10.1021/acs.jcim.4c01698

**Published:** 2025-03-26

**Authors:** Martina Rajić, Jernej Stare

**Affiliations:** Theory Department, Laboratory for Computational Biochemistry and Drug Design, National Institute of Chemistry, Hajdrihova 19, SI-1000 Ljubljana, Slovenia

## Abstract

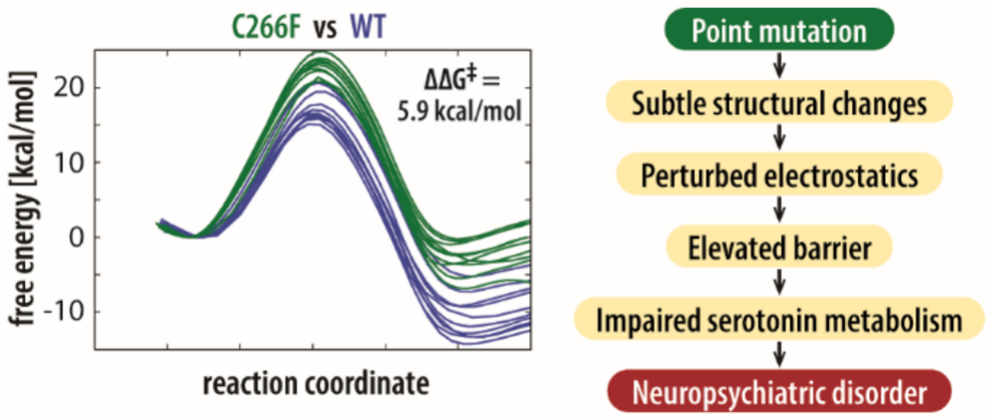

Brunner syndrome is a rare genetic disorder characterized by impulsive aggressiveness and intellectual disability, which is linked to impaired function of the monoamine oxidase A (MAO-A) enzyme. Patients with specific point mutations in the *MAOA* gene have been reported to exhibit these symptoms, along with notably elevated serotonin levels, which suggest a decreased catalytic performance of the mutated MAO-A enzymes. In this study, we present multiscale molecular simulations focusing on the rate-limiting step of MAO-A-catalyzed serotonin degradation for the C266F and V244I variants that are reportedly associated with pathologies characteristic of the Brunner syndrome. We found that the C266F mutation causes an approximately 18,000-fold slowdown of enzymatic function, which is equivalent to a *MAOA* gene knockout. For the V244I mutant, a somewhat smaller, yet still significant 300-fold slowdown has been estimated. Furthermore, we conducted a comprehensive comparison of the impact of enzyme electrostatics on the catalytic function of the wild-type (WT) MAO-A and both aforementioned mutants (C266F and V244I), as well as on the E446K mutant investigated in one of our earlier studies. The results have shown that the mutation induces a noteworthy change in electrostatic interactions between the reacting moiety and its enzymatic surroundings, leading to a decreased catalytic performance in all of the considered MAO-A variants. An analysis of mutation effects supported by geometry comparison of mutants and the wild-type enzyme at a residue level suggests that a principal driving force behind the altered catalytic performance of the mutants is subtle structural changes scattered along the entire enzyme. These shifts in geometry also affect domains most relevant to catalysis, where structural offsets of few tenths of an Å can significantly change contribution to the barrier of the involved residues. These results are in full agreement with the reasoning derived from clinical observations and biochemical data. Our research represents a step forward in the attempts of using fundamental principles of chemical physics in order to explain genetically driven pathologies. In addition, our results support the view that the catalytic function of enzymes is crucially driven by electrostatic interactions.

## 1 Introduction

Neuropsychiatric disorders are a group of complex conditions that profoundly affect the well-being and daily life of the individuals experiencing them. Common examples of such disorders include schizophrenia, bipolar disorder, major depressive disorder, and attention deficit hyperactivity disorder (ADHD).^1^ All of these disorders share a common trait of alterations in brain function, manifested through distinct neuropathologies that result from imbalances in neurotransmitter levels and irregularities in their metabolism. Monoamine oxidases (MAOs) constitute a group of enzymes that catalyze the degradation of monoaminergic neurotransmitters, and the genes encoding two MAO isoenzymes (MAO-A and MAO-B) are situated on the X chromosome. The Brunner syndrome (OMIM No. 300615) is a recessive X-linked disorder that was initially reported by Brunner et al. in 1993, based on their observations in a sizable Dutch family. The family members exhibited X-linked border disability along with abnormal behaviors such as impulsive aggression, attempted rape, arson, and exhibitionism.^2,3^ It was proposed that the observed symptoms could be linked to MAO-A deficiency, and upon sequencing, it was found that each of the five affected men carried a mutation in the *MAOA* gene. The identified mutation c.886C>T (p.Q296)^2^ led to the substitution of a glutamine (CAG) codon with a termination (TAG) codon at position 296 of the amino acid sequence. Consequently, this genetic alteration led to the formation of a truncated form of the enzyme. Subsequently, researchers discovered additional missense mutations, including C266F,^4^ R45W,^5^ V244I,^6^ and E446K,^7^ together with a truncated variant p.S251 KfsX2.^5^ Among the MAO-A variants mentioned earlier, the c.797_798delinsTT (p.C266F) variant was identified by Piton et al.^4^ in a boy with autism spectrum disorder, attention deficit, and autoaggressive behavior. His two maternal uncles who also carry the mutation, have severe intellectual disability (ID) and a history of maltreatment in early childhood. Additionally, Palmer et al.^5^ reported on a family with the missense variant c.133C>T (p.R45W), where affected males exhibited borderline to mild ID, attention deficit disorder, and limited social connections. One individual had a childhood history of explosive aggression and experienced intermittent symptoms such as flushing, headaches, and diarrhea. Furthermore, Popp et al.^6^ discovered the variant c.730G>A (p.V244I) through high-throughput sequencing in a 7-year-old boy with speech delay, moderate ID, and behavioral anomalies. Alongside the clinical symptoms, the biochemical analysis of MAO-A substrates and metabolites in patient’s plasma and urine indicated notable alterations in the breakdown of serotonin and catecholamines. The reduced activity of MAO-A leads to elevated serotonin levels, which may occur even during prenatal stage, causing changes in neuroplasticity that cannot be effectively addressed with pharmacological treatment. Based on these observations, it is believed that impaired catalytic activity of MAO-A enzyme may be a likely underlying cause for a significantly altered metabolism of neurotransmitters. Thus, these mutations deserve more thorough analysis, as the individuals having them suffer from moderate to severe neurodevelopmental disorders.

Approaching the subject from a chemical physics standpoint, the point mutations discovered in MAO-A offer a promising foundation to thoroughly explore potential changes in the enzyme’s catalytic performance. Given that electrostatic interactions are believed to play a crucial role in substrate binding, catalytic activity, and overall enzyme stability, the substitution of one amino acid to another can lead to notable changes in or around the enzyme active site. Consequently, this affects the enzyme’s ability to properly bind substrates or catalyze reactions, possibly leading to altered enzyme function or even complete loss of activity.

**Figure 1:**
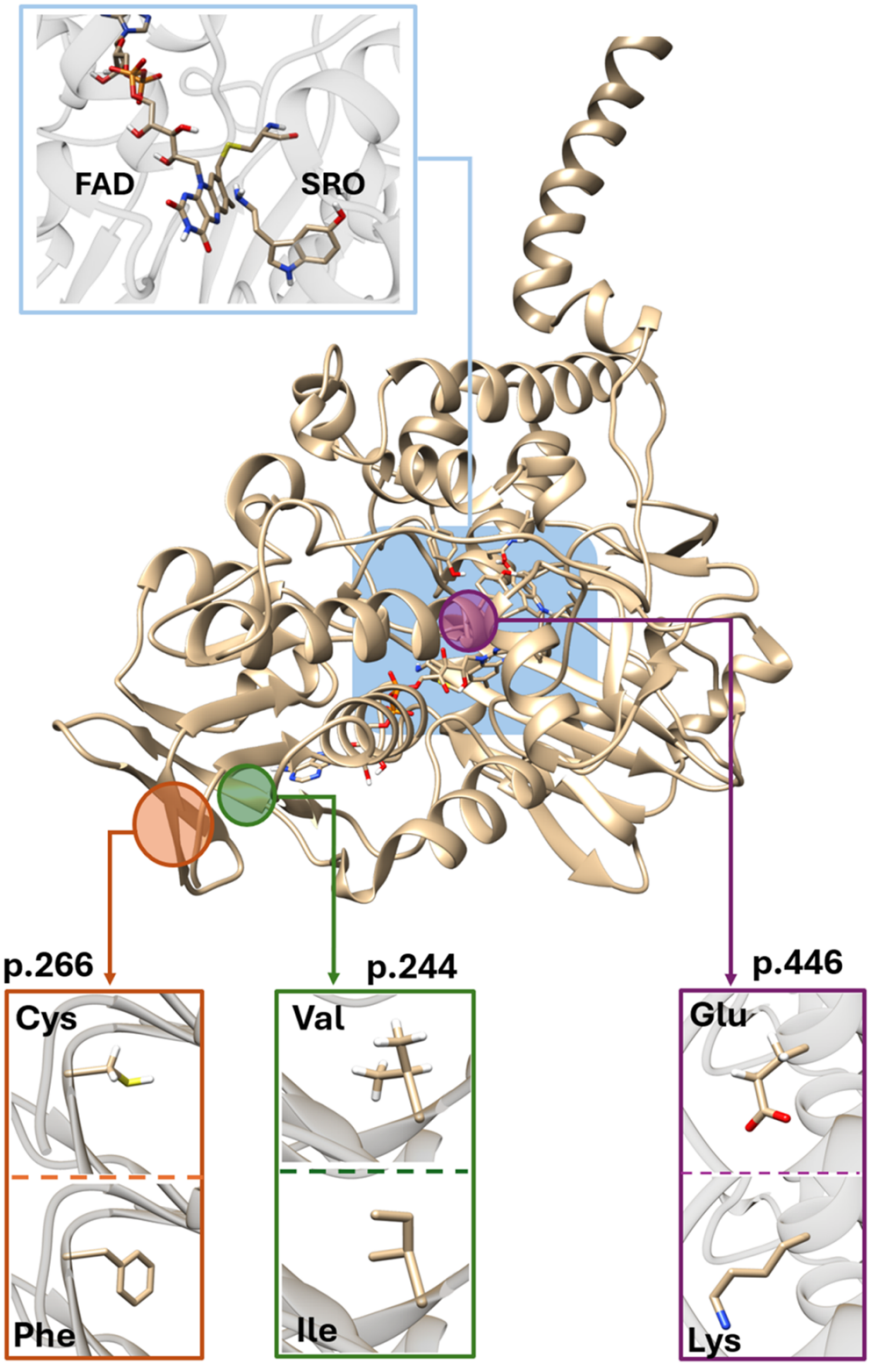
Monoamine oxidase A (MAO-A) enzyme with the location of the active site (blue shade) with the flavin adenine dinucleotide cofactor (FAD) and substrate serotonin (SRO) indicated, together with mutated residues at positions p.266, p.244, and p.446 marked with orange, green, and purple squares, respectively. The mutations are shown below where cysteine is replaced with phenylalanine for C266F, valine with isoleucine for V244I, and glutamine with lysine for the E446K variant. The mutations were conducted on the previously studied wild-type MAO-A enzyme (acquired from the Protein Data Bank under ID 2Z5X) by means of the Chimera program.

The substitution of a polar cysteine residue with a hydrophobic phenylalanine (C266F) (see Figure 1) or the replacement of negatively charged glutamate for a positively charged lysine (E446K) is expected to significantly alter the electrostatic interactions in the surroundings of the mutated residue and also over a broader spatial range. Interestingly, the substitution of valine with isoleucine (V244I) has been found to lead to the Brunner syndrome, although these two nonpolar amino acids differ by only one methyl group. These findings indicate that changes in the electrostatic environment are not solely due to change in the residue charge (e.g., negative to positive) but also result from structural modifications caused by the mutation and consequently, the unique interactions of each residue within the enzyme. Considering the proposed hypothesis that enzyme catalysis is predominantly regulated by electrostatic interactions (referred to as preorganized electrostatics),^8−12^ the substitution of one residue to another is likely to induce significant electrostatic perturbations, potentially resulting in an impaired substrate metabolism and catalytic efficiency of the MAO-A enzyme.

**Figure 2:**
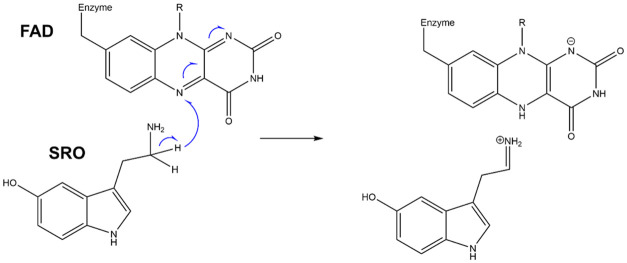
Rate-limiting step of MAO-catalyzed serotonin degradation involves a hydride transfer reaction between the reactive carbon of serotonin (SRO) and the nitrogen atom of the flavin adenine dinucleotide (FAD) cofactor within the MAO active site.

These aspects can be investigated by simulation tools tuned for the treatment of chemical reactions embedded in a complex enzymatic environment. One such approach is the established empirical valence bond (EVB) method^13−15^ that has been used in many studies of enzymatic reactions,^16−19^ including those of MAO enzymes that have been focused upon by our research group. Given that the mechanism of the rate-limiting step in the decomposition of monoamine neurotransmitters is well-known (see Figure 2),^20,21^ we were able to reliably estimate the reaction free energy barrier by EVB and convert it to the reaction rate constant using Eyring–Polanyi equation,^22^ which allows us to compare the reaction kinetics of the MAO-A mutants and of the wild-type (WT) MAO-A enzyme. On the basis of EVB methodology, we accurately investigated the catalytic performance of MAO enzymes, focusing on the effects of artificially designed MAO-A and MAO-B point mutations (i.e., mutants without known neuropsychiatric impact)^23−25^ and substrate selectivity.^26−28^ In addition to that, we addressed two MAO-A mutants known to be associated with the Brunner syndrome, namely, R45W and E446K, and found a significant slowdown of serotonin degradation^29^ compared to the WT enzyme, which is in agreement with the clinical picture of the patients carrying these mutations.

**Figure 3:**
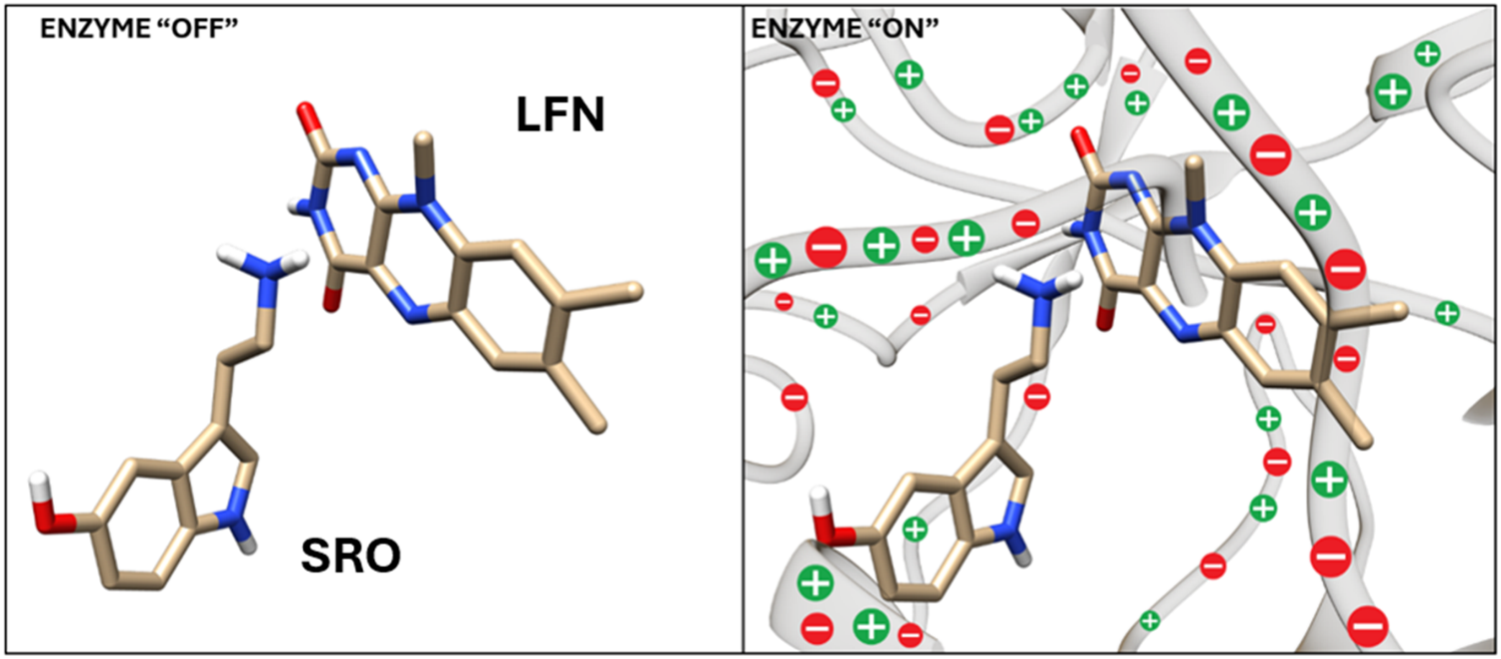
Role of electrostatics in enzyme catalysis is assessed by our computational methodology based on embedding the reacting moiety consisting of the lumiflavin part of the FAD cofactor (labeled as LFN) and serotonin substrate (labeled as SRO) (drawn as sticks) in the enzymatic surroundings represented by atomic point charges.^30^ By treating the reacting moiety with quantum chemistry (e.g., density functional theory (DFT)) either in the presence or absence of enzyme’s point charges, one can evaluate the contribution of enzyme electrostatics to the barrier lowering and elucidate subtle differences between similar enzymes (e.g., WT vs mutants). In both frames, the molecule in the bottom left of the frame is serotonin, whereas the triple ring molecule is the flavin prosthetic group.

Another viewpoint offering insight into the changed catalytic performance of an enzyme on point mutation is related to the proposed hypothesis of preorganized electrostatics.^8−12^ We developed a computational method for investigation of the impact of electrostatics in the catalytic function of enzymes.^30^ The method is based on embedding the reacting moiety treated by quantum chemistry into the enzymatic environment represented by point charges (Figure 3). These charges can be either included (enzyme “ON”) or omitted from the calculation (enzyme “OFF”), and any difference in quantities derived from quantum calculations (e.g., reaction barrier) between the ON and OFF states is attributed exclusively to the electrostatic environment provided by the enzyme. By doing so, we can directly evaluate the barrier lowering due to enzyme electrostatics, thus evaluating the underlying hypothesis on the source of catalysis in enzymes. In addition to that, one can elucidate not only the substantial barrier lowering due to electrostatics but also subtle performance difference between similar enzymes. In this way, we demonstrated that electrostatic interactions contribute to a barrier lowering of approximately 14 kcal/mol in WT MAO-A,^30^ and, in agreement with experimental observations, that the electrostatic environment of MAO-B is slightly more catalytic for phenylethylamine degradation than that of MAO-A.^31^ Also, electrostatics in the R45W (Brunner-type) mutant of MAO-A appears to be slightly less catalytic for serotonin decomposition than in the WT enzyme,^29^ further confirming the higher barrier pertaining to the mutant (as computed by EVB) and highlighting the role of electrostatics in enzymatic reactions.

In this study, our primary objective was to elucidate the catalytic performance of selected clinically relevant missense variants of monoamine oxidase A (MAO-A) toward serotonin degradation. Our aim was to obtain a more nuanced understanding of the Brunner syndrome through the application of advanced physical chemistry techniques. We utilized two distinct computational methods to systematically compare the catalytic efficiencies of the wild-type MAO-A enzyme with its missense variants, specifically C266F, V244I, and E446K. In the first part of our treatment, we simulated the reaction dynamics and evaluated the corresponding free energy profiles by EVB methodology for the WT enzyme as well as for the mutants, while in the second part we analyzed the role of electrostatics in catalytic efficiency of the enzyme (WT and mutants). By comparing the free energy barrier and the contribution of electrostatics to the catalytic performance of the enzyme between the variants, we assessed the performance of the MAO-A mutants. Because the clinical picture of the Brunner syndrome includes impaired metabolism of serotonin, it is expected that the catalytic performance of the mutants is reduced in comparison to WT MAO-A. This was already confirmed in our previous study for the R45W mutant both by EVB simulation as well as by analysis of electrostatics, whereas for the E446K mutant only EVB evaluation was performed leaving aside analysis of electrostatics.^29^ The present study increases the investigated pool of clinically relevant MAO-A mutants and validates the hypothesis that the observed mutations generally lead to deficient catalytic function of MAO-A. In addition to the C266F and V244I mutants that have not been considered by any of previous studies, we herein also include evaluation of electrostatics in the catalytic performance of the E446K mutant, which has not been carried out yet.

It is noteworthy that until now, experimental studies on the Brunner syndrome have primarily focused on investigating changes in metabolite levels in plasma or urine, caused by different mutations in the *MAOA* gene sequence.^2,4,5^ Detailed kinetic or mechanistic studies have been lacking, possibly due to the time-consuming and costly nature of experimental procedures. Here, simulation tools offer an affordable and viable platform for in-depth insight into a rather complex problem of elucidating physical aspects of a chemical reaction in various enzymatic environments that differ only slightly between each other. Due to the limited application of experimental methods for investigating electrostatic interactions within enzymes and their impact on enzyme catalysis, computational approaches remain crucial in this area of research.^17,32−38^ Hence, this study fills a critical gap between chemical physics, genetics, and clinical manifestation. Additionally, it investigates the presumed vital role of electrostatics in enzyme catalysis.

## 2 Computational Details

### 2.1 Molecular Dynamics (MD)/EVB Simulations

The WT MAO-A model was constructed on the basis of enzyme’s crystal structure available in the Protein Data Bank (PDB ID: 2Z5X).^39^ The preparation of the enzyme for the simulations followed the method described in our previous research on wild-type serotonin (SRO) degradation.^40^ MAO-A variants (mutants) were prepared by changing specific residues using the UCSF Chimera program,^41^ while all of the other conditions remained consistent with those in the wild-type study.^40^ Specifically, the following residues were changed, one at a time: (i) Glu446 was changed to Lys446 for E446K, (ii) Cys266 was changed to Phe266 for C266F, and (iii) Val244 was changed to Ile244 for the V244I variant.

The simulation model was created using the OPLS-AA force field^42^ and involved a spherical cell with a radius of 50 Å, centered on the N5 atom of the flavin cofactor covalently bound to the Cys406 residue. The cell encompassed the enzyme–substrate complex along with approximately 14,850 water molecules. Ligand parameters were obtained through the ffld_server utility with support from the Maestro v.13.6 graphical interface.^43^ Atomic charges of the ligand were computed by fitting to the electrostatic potential calculated at the HF/6-31G(d) level of theory, following the RESP scheme as implemented in AmberTools21.^44^

To prepare the system for simulations, a relaxation and equilibration process was conducted in several stages. The time-step was gradually increased from 0.1 to 1 fs, and the temperature was raised from 1 to 300 K. Positional restraints were gradually reduced during the equilibration phase. In total, equilibration required approximately 2 ns of molecular dynamics (MD). The adequacy of the relatively short equilibration was later on validated by extending the equilibration stage to approximately 20 ns, revealing no significant change in the root mean square deviation (RMSD) profiles relative to the original equilibration period (see Figure S1). Once the system reached an equilibrated state, 10 independent replicas were generated by randomizing the velocities and further equilibrating the system for an additional 100 ps. All of the subsequent steps described below were performed in a batch of 10 replicas to ensure better sampling of the reaction phase space.

For the simulation of the reactive step, a standard empirical valence bond (EVB) procedure^13−15^ was employed, which is based on the free energy perturbation/umbrella sampling^45,46^ approach. Using the coupling parameter λ, the force field of the reactants (ε_R_) was gradually transformed into the force field of the products (ε_P_) through 51 consecutive mapping steps (the change in λ between two consecutive steps was 0.02). This transformation was achieved via a mapping potential of the following type
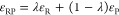
The simulations for each mapping step were conducted over a duration of 100 ps, resulting in a total of 5.1 ns of MD simulation per replica. The free energy profiles were obtained using the same EVB parameters as those acquired in the study of serotonin decomposition by MAO-A.^40^ These parameters include the EVB-specific off-diagonal matrix element of 44.28 kcal/mol and the gas-phase origin shift between the two force fields of 103.94 kcal/mol. Then, for each variant of MAO-A, the free energy barrier was determined as average over the replicas, and the impact of point mutation on reaction kinetics was evaluated by using the Eyring–Polanyi equation
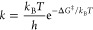
where *k* is the rate constant, *k*_B_ and *h* are the Boltzmann and Planck constants, respectively, *T* is the absolute temperature, whereas Δ*G*^‡^ denotes the free energy barrier. In the context of comparison between the WT enzyme and the mutant (MUT), the ratio of the respective rate constants *k*_WT_/*k*_MUT_ is expressed as
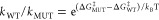
where Δ*G*_WT_^‡^ and Δ*G*_MUT_^‡^ correspond to the free energy barrier for the reaction in the WT enzyme and in the mutant, respectively.

All simulations of the reaction dynamics were carried out using the Q5 program package.^47^ For the visualization of trajectories and analysis of the simulation data, the VMD program package was utilized.^48^

Note that the EVB reaction simulation of WT MAO-A and the E446 K mutant has already been performed in earlier studies,^29,40^ but in the present case, a considerably larger simulation cell (50 Å radius compared to 30 Å) has been used. The larger cell was employed to avoid potential boundary issues because in the case of C266F and V244I mutants, the mutation point is topologically quite close to the boundary of the originally sized cell. Thus because of the specific requirement for the cell size, EVB simulations were performed for all variants of MAO-A (WT, E446 K, C266F, and V244I) to ensure comparability. A common practice with spherical simulation cells has been to set any ionizable residues close to the sphere boundary (typically within 3 Å) to a neutral (nondissociated/uncharged) state to avoid numerical instabilities during the simulation.^29,40^ Herein, when the original simulation cell is enlarged from 30 to 50 Å, those residues have been set to an ionized protonation state corresponding to physiological conditions. Only few ionizable residues in the MAO-A “tail” (three lysines altogether) remained in the neutral protonation state due to the proximity to the sphere boundary.

### 2.2 Evaluation of the Role of Electrostatics

The examination of the influence of the electrostatic environment in both the WT and the variants (E446K, C266F, and V244I) was conducted using our computational model, which has been briefly presented in the Section 1 (see Figure 3 and the related text) and extensively described in our previous studies.^29,31,49^ In this analysis, the reacting moiety consisting of serotonin (SRO) and the truncated flavin cofactor lumiflavin (LFN), was treated with quantum calculations at the M06-2*X*/6-31+G(d,p) level. On the other hand, the remaining solvated enzyme was represented by point charges identical to the ones used in the classical simulation. This representation ensures that the interactions between the reacting moiety and its surrounding environment are solely driven by Coulombic forces.

Consequently, through quantum computations conducted in both the presence and absence of point charges (enzyme ON/OFF), we can assess the impact of the electrostatic environment on the energy barrier between the state of reactants and the transition state. To implement this approach, characteristic structures of the system, known as “snapshots”, were obtained in the state of reactants (R) and in the transition state (TS), as these states are critical for assessing catalysis. For all of the variants (E446K, V244I, C266F) together with the WT enzyme, these snapshots were derived from the MD simulation presented above. In total, 200 snapshots (100 for R and 100 for TS) were extracted from the simulations for each variant of the enzyme. For each snapshot, the energy and electronic structure of the system were computed in the presence and absence of approximately 52,800 point charges representing the surrounding protein and water molecules (the number of employed point charges slightly differs between MAO-A variants). All quantum computations were carried out using the Gaussian16 program package.^50^

To further examine the role of electrostatics and the mechanism impacting the enzyme performance due to mutations, we extended the above-presented approach to study the contribution of individual residues, one at a time, by performing semiempirical PM6^51^ single-point calculations on the same snapshot structures, but this time in the presence of point charges pertaining to a single given residue. 512 MAO-A residues were individually considered in this way for all MAO-A variants, totaling in (512 residues) × (200 snapshots per enzyme variant) × (4 enzyme variants) = 409,600 single-point calculations. Such a massive amount of calculations would have not been practical with the DFT methodology that has been applied herein to the solvated enzyme as a whole, but the low-cost PM6 treatment is feasible for this purpose, albeit at the expense of accuracy (see below). Alongside with this treatment, we also analyzed the snapshot structures in terms of average distances between individual residues and the reacting moiety. We computed these distances by averaging, for each snapshot structure, positions of all atoms of the reacting moiety as well as of the corresponding residue, computed the distance between these positions, and averaged it over all snapshots. We carried out this separately for all four MAO-A variants, facilitating a geometry comparison between them.

## 3 Results and Discussion

### 3.1 EVB Simulations

**Figure 4:**
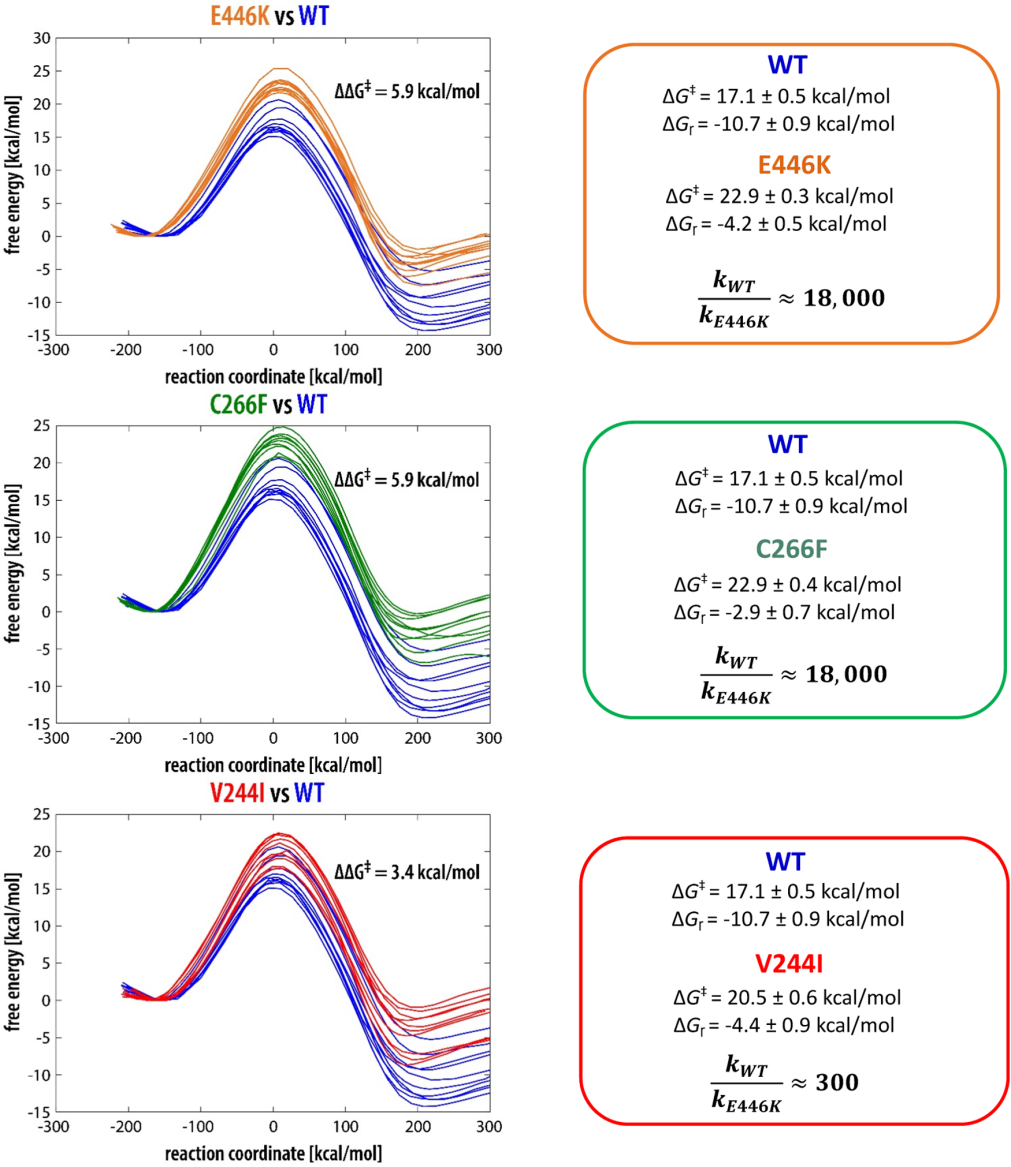
Pairwise comparison of free energy profiles for the rate-limiting step reaction involving serotonin and the flavin cofactor between three mutated versions of MAO-A and the wild-type (WT) enzyme (from top to bottom: E446K, C266F, and V244I). The profiles are depicted in blue for the WT, in orange for E446 K, in green for C266F, and in red for the V244I mutant. Additionally, the free energy barrier and the reaction free energy are provided for both the WT and the variants (E446K, C266F, and V244I), along with their respective standard errors of the mean (SEM). The ratio of the corresponding rate constants derived from the Eyring–Polanyi equation are also displayed.

Free energy profiles of the rate-limiting step of serotonin oxidation computed by EVB simulations for WT MAO-A and its mutants E446K, C266 V, and V244I are displayed and compared in Figure 4 together with the associated free energy barriers. For each variant of the enzyme, the profiles have been computed in a batch of ten replicas differing slightly in initial conditions and the displayed barrier corresponds to an average over the replicas with the standard error of the mean given in parentheses. The strategy of performing the simulation in replicas allows for an improved sampling of the reaction phase space, which can be done in parallel (rather than running a single but several times longer simulation), thus shortening the required time. For WT MAO-A, the EVB barrier is estimated to 17.1 kcal/mol. This value is in excellent agreement with experimental kinetic measurements of serotonin degradation by MAO-A yielding the rate constant of 67.4 min^–1^,^52^ which converts to a barrier of 17.5 kcal/mol. Note that the experimental barrier is available only for WT MAO-A but for none of the herein considered mutants.

In comparison to the WT enzyme, all of the mutated variants feature a noticeably higher barrier. The barrier computed for the E446K variant is 22.9 kcal/mol, which is by 5.9 kcal/mol higher than for the WT. For this particular mutant, the barrier change relatively to WT has already been estimated in our earlier study, yielding a somewhat smaller increase of the barrier, namely, by 3.6 kcal/mol.^29^ The slight difference in prediction between this and previous study can likely be attributed to the slightly different simulation setup in the present study, which among the rest uses a larger simulation sphere for all enzyme variants. Nevertheless, in either case, the barrier increase in E446K appears to be significant and of sufficient magnitude to cause a substantial slowdown of serotonin metabolism, the previously and presently estimated reduction in the rate constant amount to approximately 450 and 18,000, respectively. In both cases, the estimated slowdown is essentially equivalent to a *MAOA* gene knockout. Note that the apparently large difference between both estimates is due to the exponential relation between the barrier and the rate constant, in which even small barrier differences are largely amplified by conversion to reaction rates.

In a very similar fashion, both C266F and V244I mutants feature an increased barrier relative to WT (Figure 4). For C266F, the EVB-computed barrier amounts to 22.9 kcal/mol and is (by chance) virtually identical to the one computed for the E446K mutant, hence the serotonin oxidation slowdown for C266F and E446K is identical, namely, 18,000-fold. On the other hand, the barrier increase in V244I is slightly smaller, by 3.4 kcal/mol relative to WT, but this still converts to substantially slower kinetics (an approximately 300-fold slowdown). Therefore, the present calculations suggest drastically impaired serotonin metabolism in all three mutated variants of MAO-A. This is in agreement with clinical observations associated with the investigated mutants, as well as with the analyses of metabolites, all of which suggesting elevated serotonin levels in the central nervous system and likely deficient performance of mutated MAO-A variants in affected patients.^2,4,5^

For each variant of MAO-A, the free energy profiles displayed in Figure 4 feature deviations between individual replicas. These deviations can be attributed to limitations associated with sampling of the reaction phase space. Because of the complexity of the phase space present already within the reacting moiety, relatively long simulation times (microseconds and longer) are required to entirely diminish the differences between the replicas even in the gas phase,^53^ let alone within the enzymatic environment. Therefore, obtaining fully convergent free energy profiles for the reaction in question is beyond the available resources. The present strategy based on replicas definitely enhances the sampling, but the cumulative simulation period of 51 ns (for each MAO-A variant) evidently retains traces of incomplete sampling, as reflected in the profile differences between the replicas. In Figure 4, variations in the barrier and free energy of the reaction are quantified by their respective standard errors of the mean (SEM), indicating sensitivity of the average barrier and reaction energy to variations between the replicas. The SEM of the barrier is in the range between 0.4 and 0.6 kcal/mol and is several times smaller than the observed barrier elevation due to mutation (3.4–5.9 kcal/mol). Therefore, the estimated decreased performance of mutated MAO-A with respect to the WT variant is significant, even if profile variations are considered.

### 3.2 Role of Electrostatics in the Impaired Performance of MAO-A Mutants

**Figure 5:**
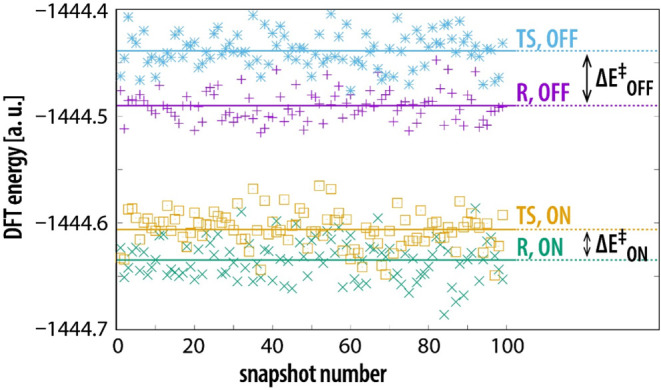
Energies of snapshots representing the reactant state (purple points/R, OFF) in the absence of enzyme electrostatics; the transition state in the absence of enzyme electrostatics (blue points/TS, OFF); the reactant state in the presence of enzyme electrostatics (green points/R, ON); and the transition state in the presence of enzyme electrostatics (yellow points/TS, ON). Colored horizontal lines denote the average energy of the corresponding set of snapshots. Selected energy differences are labeled as follows: Δ*E*_OFF_^‡^—reaction barrier in the absence of enzyme electrostatics; Δ*E*_ON_^‡^—reaction barrier in the presence of enzyme electrostatics.

The significant increase in the free energy barrier on herein considered MAO-A point mutations reveals a deficient catalytic performance caused by mutation. We further addressed the observed performance decrease in light of the popular hypothesis that enzyme catalysis is mainly governed by electrostatic interactions.^12^ For each variant of MAO-A, we extracted from the present FEP/EVB simulation trajectories snapshot structures representative of R and TS stages of the reaction (100 snapshots for each of the two). These stages are essential for the evaluation of kinetics and catalysis. The reacting moiety consisting of serotonin and flavin was treated by DFT whereas the surrounding atoms of enzyme and water molecules were represented by atomic point charges taken from the classical simulation. For each of these structures, we performed two single-point DFT calculations, one including the surrounding charges (enzyme ON) and the other without charges (enzyme OFF). In this way, the difference between the ON and OFF states is exclusively due to electrostatics originating from the surrounding enzyme. By comparing the energy difference between the TS and R states (averaged over the snapshots) in the presence and absence of enzyme point charges, one can directly evaluate the impact of electrostatics on the barrier and thus elucidate the potential catalytic function of enzyme electrostatics. Energetics of snapshots of WT MAO-A is presented in Figure 5.

The electrostatic environment provided by the enzyme stabilizes both R and TS, but the latter is stabilized to a greater extent, meaning that the enzyme electrostatics plays a catalytic role. Specifically, for WT MAO-A, the barrier computed for the OFF state (Δ*E*_OFF_^‡^) is 32.3 kcal/mol, whereas on inclusion of enzyme electrostatics, the barrier drops to 17.9 kcal/mol (Δ*E*_ON_^‡^). The barrier difference between the OFF and ON state is 14.4 kcal/mol in favor of the latter, which can be attributed to the catalytic effect originating from enzyme electrostatics. These values are in very good agreement with previously published DFT calculations of the reaction mechanism,^20,23^ and the estimated barrier with included enzyme electrostatics is in an excellent match even with the experimental data. However, previous experience suggests that slight mismatch in barriers evaluated by the EVB approach on one side and our charge-embedding treatment on the other may emerge due to discrepancies between the force field used in classical simulations from which the snapshots are extracted on one side and the DFT potential energy surface on the other;^30^ therefore, the present good agreement may be fortuitous. Nevertheless, the magnitude of the catalytic effect of 14.4 kcal/mol firmly demonstrates that electrostatics plays a dominant role in the catalytic function of WT MAO-A, as has been shown in our earlier study, yet with a slightly different substrate phenylethylamine.^30^

The mutated variants of MAO-A feature a very similar qualitative pattern of energetics of the R and TS in the presence/absence of enzyme electrostatics as the WT enzyme (Figure 5). However, characteristic energies differ among the variants, yielding different estimates of the catalytic effect due to electrostatics. Table 1 lists the evaluated catalytic effect, as well as the barriers in the presence and absence of electrostatic environment for all considered MAO-A variants.

**Table 1:** Barriers Computed in the Absence (Δ*E*_OFF_^‡^) and Presence of the Enzyme’s Electrostatic Environment (Δ*E*_ON_^‡^) for WT MAO-A and Its Mutants^a^

MAO-A variant	Δ*E*_OFF_^‡^	Δ*E*_ON_^‡^	ΔΔ*E*_ON–OFF_^‡^
WT	32.3	17.9	–14.4
E446 K	32.9	20.6	–12.3
C266F	35.4	23.3	–12.1
V244I	30.5	24.4	–6.1

a The difference in the barrier between the OFF and ON states (ΔΔ*E*_ON–OFF_^‡^) is a direct measure of the catalytic effect due to enzyme electrostatics and is also listed. All of the quantities are derived from the corresponding energies averaged over 100 snapshots, representative of the reactant and transition state (see Figure 5). All quantities are in kcal/mol.

In all cases, the barriers feature a significant drop in inclusion of enzyme electrostatics in the calculation (all Δ*E*_ON_^‡^ values are smaller than their Δ*E*_OFF_^‡^ counterparts, so that all ΔΔ*E*_ON–OFF_^‡^ values are negative). This confirms electrostatic interactions to play a vital role in the catalytic function of all of the herein considered variants of the enzyme. Notably, in all of the mutants, the barrier lowering (ΔΔ*E*_ON–OFF_^‡^) is smaller than in the WT enzyme, suggesting that the catalytic function of MAO-A is reduced on mutation largely owing to electrostatics effects. In addition, the barriers in the presence of surrounding charges (Δ*E*_ON_^‡^) are significantly higher in the mutants than in the WT enzyme, further confirming the trend.

The evaluated gas-phase barriers (Δ*E*_OFF_^‡^) span the range between 30.5 and 35.4 kcal/mol, which is a noteworthy variation; however, one should mind that in the present treatment, the geometries of the reacting moiety have been extracted from a simulation of the fully scaled enzyme reaction and are therefore “biased” by the surroundings, which in principle reflects also variations related to mutation. Therefore, variations in Δ*E*_OFF_^‡^ can be expected, but a detailed analysis of their magnitude is beyond the scope of the present work. Nonetheless, all of the tabulated gas-phase barriers are in reasonable agreement with previous studies of the reaction and its mechanism by using regular quantum chemistry protocols (transition-state optimization, etc.) and the same or very similar level of treatment.^20,53,54^

While the Δ*E*_ON_^‡^ and ΔΔ*E*_ON–OFF_^‡^ values are in very good qualitative agreement with the EVB simulations, fine details such as precise barrier elevation relative to WT, or the barrier elevation trend among the mutants, do not provide a quantitative match. For example, the E446K and C266F mutants feature virtually identical EVB-computed barrier of 22.9 kcal/mol, whereas with our charge-embedding model, they have been estimated to 20.6 and 23.3 kcal/mol, respectively. At the same time, the EVB barrier for the V244I mutant has been evaluated to 20.5 kcal/mol, whereas our model yields a value of 24.4 kcal/mol. Considering also variations in Δ*E*_OFF_^‡^, the barrier lowering due to electrostatics (ΔΔ*E*_ON–OFF_^‡^) appears to be half smaller for V244I than for other two mutants (6.1 vs slightly above 12 kcal/mol). These notable but relatively minor discrepancies are understandable and can likely be attributed to fundamental differences between EVB and our treatment, in that the former includes the full array of nonbonding interactions whereas the latter uses only electrostatics; also, EVB simulation delivers free energy profiles (with thermal fluctuations and entropic effects included), while our treatment addresses to this only to a limited extent by averaging over snapshots, thereby remaining on potential rather than on the free energy surface. Also, the rate of sampling is drastically different between EVB and our present treatment in that the EVB profiles have been constructed on the basis of millions of energetics data points acquired during MD, whereas our treatment relies on a several orders of magnitude smaller data set consisting of 200 data points (100 for R and 100 for TS). Taking into account these factors, results obtained by both approaches are in fine agreement, despite some differences. It may be assumed though that our charge-embedding scheme can hardly discern between barriers differing by less than ∼1–2 kcal/mol, rendering the assessment of very subtle differences difficult. Nevertheless, the presently observed differences in barriers within the enzyme’s electrostatic environment (Δ*E*_ON_^‡^) between WT MAO-A and mutants are large enough to firmly support the reduced catalytic power in all mutants, as compared to WT.

### 3.3 By-Residue Analysis of Catalytic Performance and Geometry

In our previous work, we have been able to rationalize the factors governing the point mutation effect even at a residue level.^29^ This has been done by scrutinizing the interaction between the dipole moment of the reacting moiety and the electric field cast by its enzymatic surroundings. As the electric field is formally additive at the point charge (atomic) level and therefore also at the residue level, such a representation allows for a by-residue analysis of catalytic performance of an enzyme. However, in the present work, attempts at using this approach did not yield convincing results, possibly due to limited validity of representing the reacting moiety solely by its dipole moment vector. This issue requires further investigation, but that is beyond the scope of the present work. Consequently, we analyzed the interactions of individual residues with the reacting moiety by embedding the latter in the pool of the surrounding point charges of individual residues one at a time. In essence, this treatment is equivalent to embedding the reacting moiety (treated by DFT) into its entire enzymatic surroundings presented and discussed above. However, due to the huge number of single-point calculations required to consider all residues of all variants of MAO-A (over 400,000 calculations), we applied a cost-efficient semiempirical quantum method PM6 rather than DFT. In this way, contribution of electrostatics cast by individual residues to the barrier has been evaluated and compared between the enzyme variants.

First, we found that the vast majority of MAO-A residues, irrespective of the enzyme variant, exhibit a very low or vanishingly small contribution to the reaction barrier. Over 90% of residues contribute to the barrier less than |0.1| kcal/mol, and the net effect of these residues is close to zero. Apparently, we can attribute most of the enzyme’s catalytic performance to a small amount of residues accumulated in four or five short sequence domains in which individual contribution of a residue can reach up to |3| kcal/mol (note that the contribution of a residue can be catalytic, if electrostatics of that residue lowers the barrier, or anticatalytic if vice versa). Geometry inspection confirms that these domains are topologically close to the active site, typically at 12 Å or less. These features are in full agreement with our previous studies carried out with slightly different approaches.^29,31,49^ Contributions of individual residues along the amino acid sequence are displayed in Figure S2 for WT MAO-A, and analogous patterns corresponding to the mutants (not shown) are very similar. Accordingly, differential plots demonstrating by-residue performance differences between a particular mutant and WT MAO-A share the feature of a few catalytically significant domains, whereas most of the residues are completely insensitive to mutation (Figure S3). Location of domains of high-impact residues within the sequence is practically insensitive to mutation. Interestingly, these domains may not even include the mutation point (C266F and V244I are such cases), suggesting that factors other than plain electrostatic perturbation at the mutation point are crucial for the changed enzyme performance.

**Figure 6:**
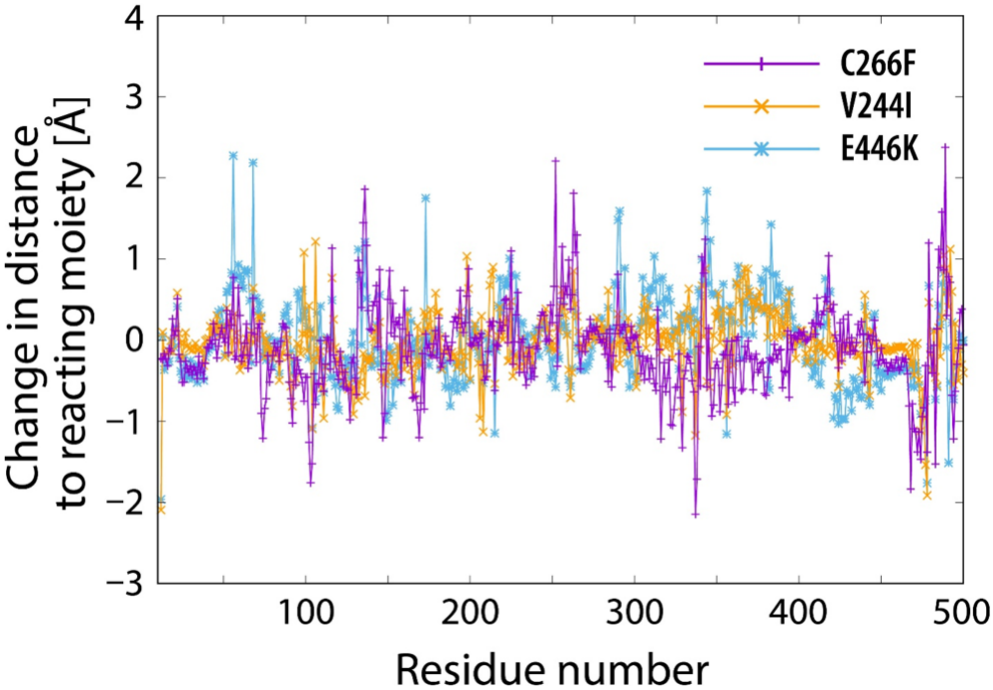
Change in distance between a residue and the reacting moiety of MAO-A on mutation (relative to the WT enzyme) along the amino acid sequence. Purple line—C266F mutant; orange line—V244I mutant; and light blue line—E446K mutant.

Next, we scrutinized the geometric features of the snapshots collected from the herein reported FEP/EVB simulation of the reactive step. We focused on the average distance between a residue and the reacting moiety and found that mutation invokes structural changes that appear to be subtle, but likely they have significant impact on the enzyme performance, as will be demonstrated below. Plots of distance change on mutation of WT MAO-A along the amino acid sequence are displayed in Figure 6.

Despite mutation affecting only one residue in the sequence, most of the residues change their positions relative to the active site (reacting moiety), as reflected in the displayed distance change profiles. While most of the changes are rather subtle, not exceeding 0.5 Å, larger shifts up to 3 Å can be observed. Apparently, in silico mutation followed by a relatively short relaxation already provides noteworthy structural shifts, which are in qualitative agreement with RMSD profiles corresponding to relaxation (Figure S1). Furthermore, profiles of distance change in mutants are radically different from one another. This confirms that enzyme point mutation has a profound and complex influence on the structure of the enzyme, reflecting a myriad of highly sensitive interactions, and suggests that establishing any correlation between the location and nature of the mutation on one side and the corresponding structural changes on the other side represents an extremely challenging task.

**Figure 7:**
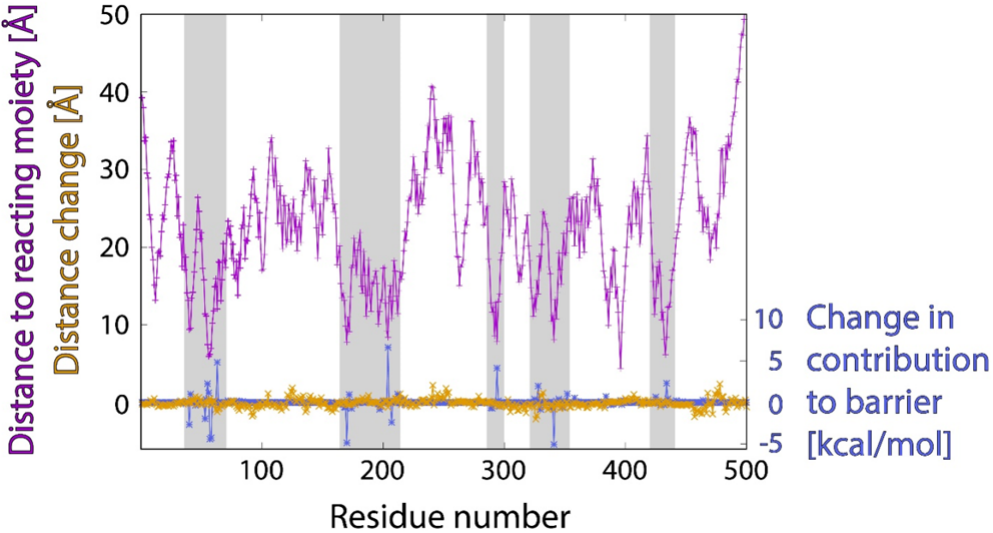
Effect of C266F mutation of MAO-A (relative to WT) along the amino acid sequence: change in contribution of the residue to the barrier (blue line and points); change in distance between the residue and the reacting moiety (orange line and points). Absolute distance (WT MAO-A) between the residue and the reacting moiety is also displayed (purple line and points). Gray shading indicates sequence domains in which the catalytic performance of the mutant is significantly altered.

But how relevant (if at all) are these shifts for the altered catalytic performance of a mutated enzyme? This can be evaluated by combining energetic and geometric features, which is displayed in Figure 7 for a selected C266F mutation.

As mentioned above, noteworthy contributions to the catalytic performance of MAO-A (and their changes on mutation; see blue peaks sticking out from the baseline) are present in five short sequence domains. Visual inspection reveals that in all of these domains, the residues are relatively close to the reacting moiety (purple profile)—often below 10 Å but rarely exceeding 15 Å. While distance offsets (orange profile) are visible along the entire sequence and are not limited to the shaded domains, they are apparently not related to altered performance when the distance between the corresponding residue and the reacting moiety is large. However, in residues close to the active site, the catalytic performance appears to be very sensitive to the geometry change; for instance, in the rightmost shaded area, the distance changes appear to be very small (mostly not exceeding 0.2 Å), yet alterations in individual catalytic performance up to 1.2 kcal/mol are observed. Likely, the altered catalytic performance is not only related to the distance between a residue and the reacting moiety but also to their orientation and other factors that have not been undertaken here due to simplicity.

The C266F case displayed in Figure 7 represents a distal mutation in which the mutation point is located far from the active site (at ∼34 Å). At ∼26 Å distance, the V244I case is similar; both mutations invoke no change in charge because all of the involved residues are neutral. On the other hand, the E446K mutation is located much closer to the active site (at ∼12 Å); furthermore, it converts the residue charge from −1 to +1 (glutamate to lysine), casting large electrostatic perturbation in the enzymatic environment. Yet, despite the considerable difference in the nature of these mutations, their corresponding by-residue profiles are fairly similar, and so are their net effects on the reaction kinetics. This implies that subtle but extremely complex structural changes smeared over the entire enzyme scaffold may be the leading factor governing the effect of point mutation on the enzyme performance, rather than possible major electrostatic perturbations emerging at the mutation site.

It should be stressed that the present by-residue analysis of contributions to the catalytic performance of MAO-A variants features considerable drawbacks related to the PM6 method. Test calculations reveal quite limited accuracy of PM6, in particular, the present treatment fails to predict the net catalytic effect of WT MAO-A because the sum of residue contributions is positive (anticatalytic). For this sake, we refrain from comparing the net effects of residues between the MAO-A variants. However, we believe the qualitative patterns of contributions of the residues to the barrier are valid among the rest due to similarity with cases previously studied by different methods.^29,31,49^

Summarizing our results, it can be concluded that the herein considered mutations of MAO-A noticeably impair the catalytic function of the enzyme, which is in full agreement with the documented (clinical) observations. At the same time, much of the decrease in performance can be attributed to electrostatic effects, thereby supporting the hypothesis that enzyme catalysis is driven by electrostatic interactions, even at the level of relatively small variations between closely related enzymes, such as mutated variants of the same enzyme.

## 4 Conclusions

We scrutinized the catalytic performance of three distinct mutants (E446K, C266F, and V244I) of the MAO-A enzyme exhibiting a neuropsychiatric clinical picture known as the Brunner syndrome in individuals bearing either mutation encoded in the genome. We did so by using two distinct computational methodologies, one being the established MD simulation based on FEP and EVB techniques and the other our own charge-embedding treatment for assessing the impact of electrostatics on the catalytic function of enzymes. The reaction subject to our investigation was oxidation of serotonin by flavin, the latter being the prosthetic group present in the active site of MAO-A. Our EVB simulations yielded free energy profiles, whereas our electrostatic treatment delivered the amount of barrier lowering due to the charged enzymatic environment. Both methodologies demonstrate significant decreases in the catalytic function in all of the mutated variants, as compared to the WT enzyme. Specifically, the EVB-computed reaction barrier was between 3.4 and 5.9 kcal/mol higher than in the WT MAO-A, giving rise to 300–18,000-fold slowdown of the rate-limiting step of serotonin oxidation, which is effectively equivalent to *MAOA* gene knockout. Consequently, these results suggest a notable rise in serotonin levels in the affected individuals, highlighting a profound disruption in neurotransmitter metabolism, which is consistent with clinical and biochemical data observed in the Brunner syndrome.^2,4,5^

In agreement with EVB results, calculations based on embedding the reacting moiety treated by DFT in a pool of point charges representing the enzymatic surroundings additionally confirm an increased reaction barrier in all mutants, as compared to the WT enzyme. Alongside that, in all mutated variants, the catalytic contribution of enzyme electrostatics appears to be smaller than in the WT enzyme. This is in agreement with the view that enzyme electrostatics not only provides major contribution to barrier lowering but is also capable of finely tuning subtle performance details, as has been already demonstrated by comparing the role of electrostatics in MAO-A and MAO-B isoenzymes.^31^ The present analysis also shows that enzyme electrostatics substantially lowers the barrier in all of the considered variants, thereby supporting the hypothesis of preorganized electrostatics as the principal driving force of the catalytic function of enzymes.

Unlike C266F and V244I variants that have not yet been investigated for their performance from the chemical physics standpoint, the E446K mutant has already been subject of a similar study^29^ but only at the EVB level. While the present EVB results confirm substantial barrier elevation for this mutant, this study also adds new insight into the function of its electrostatics, showing that all of the so far considered mutants related to the Brunner syndrome share a great deal of similarity in that they feature noticeably poorer catalytic performance in comparison to the WT enzyme.

Analysis of contribution of individual residues to the catalytic function of the enzyme assisted by the evaluation of structural changes due to mutation reveals that in each of the mutants the distance between any residue (no matter how distant from the mutation site) and the reacting moiety is subject to relatively small changes (up to 0.5 Å in most cases, but sometimes up to 3 Å) relative to the WT enzyme. Importantly, these subtle changes are present along the entire sequence, including the minor share of residues (<10%) fairly close to the active site that are most responsible for the catalytic effect. For these residues, even a shift of ∼0.2 Å may result in significant change of their contributions to the reaction barrier, implying that the slightly perturbed structure of the mutants may well be significantly less catalytic, as confirmed by EVB simulation. It is likely that these highly dislocated but subtle structural effects prevail over large localized perturbations such as changing the negatively charged residue to a positively charged one (i.e., the E446K mutation) in close vicinity of the active site, namely, distal mutations C266F and V244I void of any large charge perturbations feature a similar barrier change as E446K.

Few caveats possibly impacting the accuracy of the presently used methods should be discussed. In the first place, the sampling quality is rather limited due to relatively short simulation times both at the relaxation/equilibration level and at the level of reaction dynamics (FEP/EVB). Unfortunately, the employed program package can barely allow for much longer simulations in a reasonable time. Clearly, the use of performance-optimized program packages such as Gromacs would significantly improve the relaxation and reaction path sampling, accounting for possible structural shifts occurring at up to a microsecond time scale. Since support for EVB simulations has been recently added to Gromacs,^55^ our challenge for the future work is to critically evaluate the results of the present and earlier studies, mainly in light of the sampling quality.

In this study, our solvated enzyme model did not include any salt (counter)ions. Apart from practical reasons, we argue that the net charge of the enzyme (and thus of the entire system) was zero for the WT enzyme as well as for the C266F and V244I mutants, whereas for the E446K mutant, the net charge was +2. Although a nonneutral system may represent a source of inaccuracy, we feel that the relatively small net charge does not pose a significant impact on a system of the present size among the rest because no periodicity has been undertaken in the present model.

The PM6 semiempirical method employed herein was chosen due to CPU economy. While capable of carrying out hundreds of thousands of single-point calculations in a reasonable time, the method features inherent drawbacks, impairing the accuracy of computed energies and barriers. For this sake, PM6 calculations have been used mainly in order to demonstrate the trends in the contribution of individual residues to enzyme’s performance, but quantitative assessment of the altered catalytic power due to point mutation has been avoided. Application of superior DFT methods would likely allow for a more quantitative elucidation, but at present, such treatments are not practical.

Overall, this study represents a significant effort to integrate physical chemistry methods with genetic research to enhance our understanding of the Brunner syndrome. By focusing on the electrostatic environment of MAO-A, we aimed to provide a more comprehensive view of how enzyme function is modulated by genetic variations and how these variations impact the enzyme’s ability to process serotonin. In addition, by enlarging the investigated pool of clinically relevant mutants of MAO-A, we further validate the approaches employed. In perspective, methodologies based on chemical physics capable of accurately predicting the altered function of mutated enzymes have the potential to find application in precision medicine. In light of this, attempts will be made at further elucidating the physical background of the Brunner syndrome by addressing other emerging mutants of MAO-A. Furthermore, we plan to advance the present treatment by scrutinizing the catalytic effect of electrostatics at the level of individual residues, as we did in the past,^29,31^ as well as by including artificially designated mutants in the analysis. We expect to find a similar pattern for other point mutations, which would support the clinical picture of the Brunner syndrome. However, addressing kinetic data for all relevant mutants poses a significant future challenge, both experimentally and computationally.

## Data Availability

The structure of wild-type MAO-A utilized in this study has been taken from the RCSB Protein Data Bank database (https://www.rcsb.org/), accession code 2Z5X. Additional data generated during the research and/or analyzed in the current study are available from the corresponding author upon request.

## Supporting Information Available

RMSD profiles of enzyme relaxation and contribution of individual residues to the catalytic performance of WT MAO-A and mutants (PDF)
