# Correction to “Investigation of Electrostatic Effects on Enzyme Catalysis: Insights from Computational Simulations of Monoamine Oxidase A Pathological Variants Leading to the Brunner Syndrome”

**DOI:** 10.1021/acs.jcim.6c01268

**Published:** 2026-05-06

**Authors:** Martina Rajić, Jernej Stare

**Affiliations:** † Faculty of Pharmacy, University of Ljubljana, Aškerčeva cesta 7, 1000 Ljubljana, Slovenia

The authors wish to correct the published article with the following changes:

1. One of the two affiliations of Martina Rajić was omitted in the original publication. The additional affiliation must be listed as the primary affiliation and should read: University of Ljubljana, Faculty of Pharmacy, Aškerčeva cesta 7, 1000 Ljubljana, Slovenia.

The existing affiliation of Martina Rajić (Theory Department, Laboratory for Computational Biochemistry and Drug Design, National Institute of Chemistry, Hajdrihova 19, Ljubljana SI-1000, Slovenia) should remain unchanged.

2. Additionally, there is a typographical error in the title of the manuscript. The word “Enyzme” should be corrected to “Enzyme”. The correct title is Investigation of Electrostatic Effects on Enzyme Catalysis: Insights from Computational Simulations of Monoamine Oxidase A Pathological Variants Leading to the Brunner Syndrome.

These corrections do not affect the scientific conclusions of the paper.

