# Supplementary material for: Investigation of Electrostatic Effects on Enyzme Catalysis: Insights from Computational Simulations of Monoamine Oxidase A Pathological Variants Leading to the Brunner Syndrome
Source: J Chem Inf Model. 2025 Mar 26;65(7):3439–50. doi: 10.1021/acs.jcim.4c01698 (PMC12004519; doi:10.1021/acs.jcim.4c01698)
Supplement: Supplementary file 1 — ci4c01698_si_001.pdf [file ci4c01698_si_001.pdf]

Investigation of Electrostatic Effects on Enyzme Catalysis: Insights from Computational Simulations of Monoamine Oxidase A Pathological Variants Leading to Brunner Syndrome

*Martina Rajić, Jernej Stare**

Theory Department, Laboratory for Computational Biochemistry and Drug Design
National Institute of Chemistry, Hajdrihova 19, SI-1000 Ljubljana, Slovenia

**Corresponding author, e-mail: jernej.stare@ki.si*

SUPPORTING INFORMATION

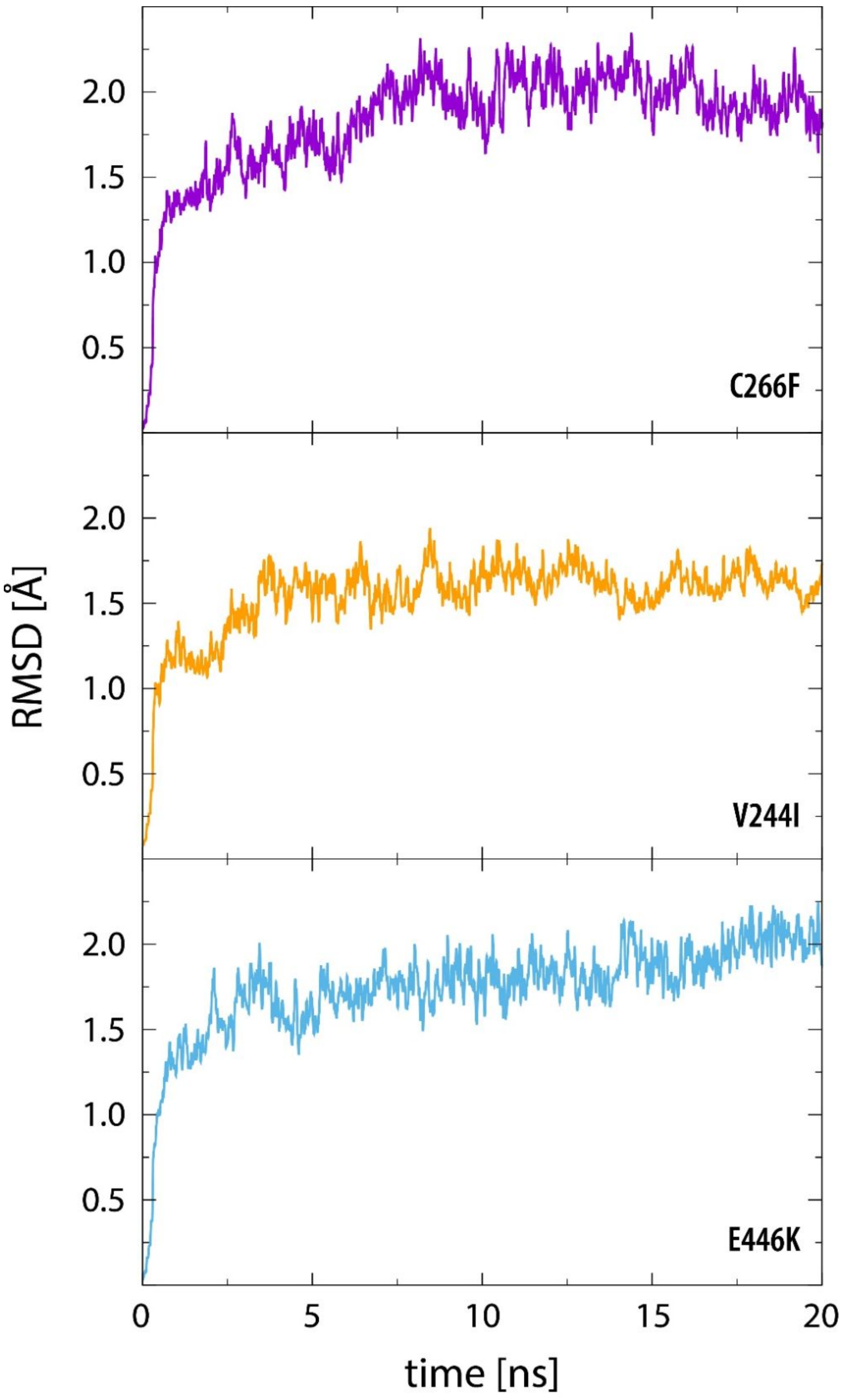

Figure S1. RMSD profiles (corresponding to the enzyme backbone) of MD relaxation of three mutants of MAO-A. All relaxations have been carried out with the Q v. 5 program (see Section 2). Subsequent FEP/EVB simulations of reaction dynamics have been performed after 2 ns of relaxation. Here, prolonged relaxations of 20 ns apparently provide some change of structures relative to initial 2 ns, but these changes appear to be of relatively small magnitude. Naturally, longer relaxations approaching microsecond regime could reveal major structural shifts of the tertiary structure of the enzyme, but that remains to be verified by further studies. Similarly, enhanced sampling would be welcome in the simulation part related to reaction dynamics, because the enzyme structure adapts to the transition state of the reacting moiety during the process. Nevertheless, RMSD profiles displayed above are in fair agreement with structural changes observed in the mutants already after short relaxation (see Section 3. 3 and Figs. 6-7.).

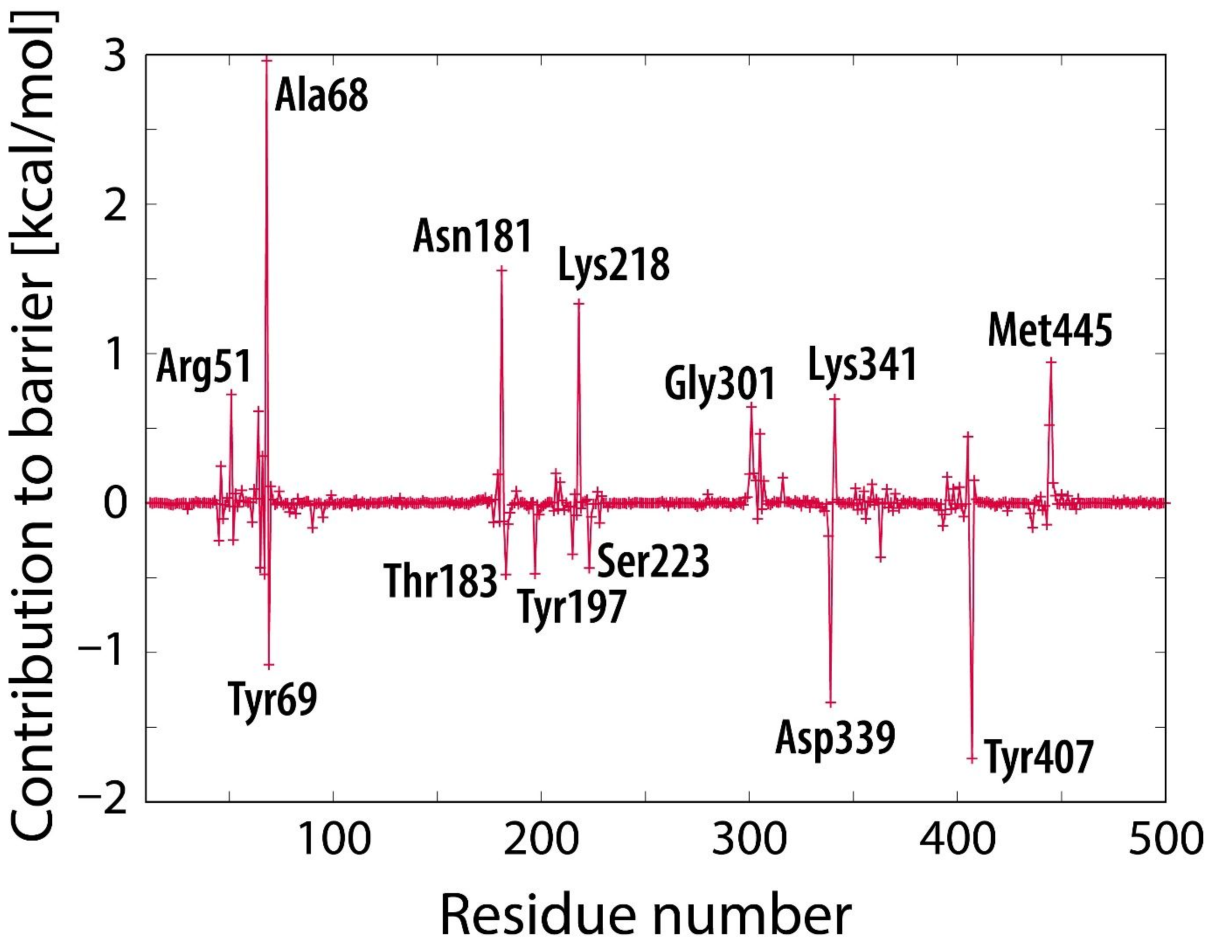

Figure S2. Contributions of individual residues of WT MAO-A to the catalytic performance of the enzyme along the sequence. A negative value corresponds to catalytic role of a given residue (barrier lowering), whereas positive contribution elevates the barrier and thus the residue acts anticatalytically. Residues with large (catalytic or anticatalytic) contributions are indicated. One can note that, while most of the residues barely affect the barrier, those with noteworthy contributions are located just in few relatively narrow sequence domains located close to the active site (see Section 3.3). The contributions have been calculated on snapshots extracted from precedent FEP/EVB simulations of reaction dynamics (see Section 2) and include 102,400 single point calculations using the PM6 semiempirical method. Note that the PM6 method, while being cost-efficient, lacks accuracy, among the rest the sum of individual residue contributions is positive (anticatalytic), which is essentially incorrect; however, the pattern of contributions over the sequence and other qualitative trends are valid.

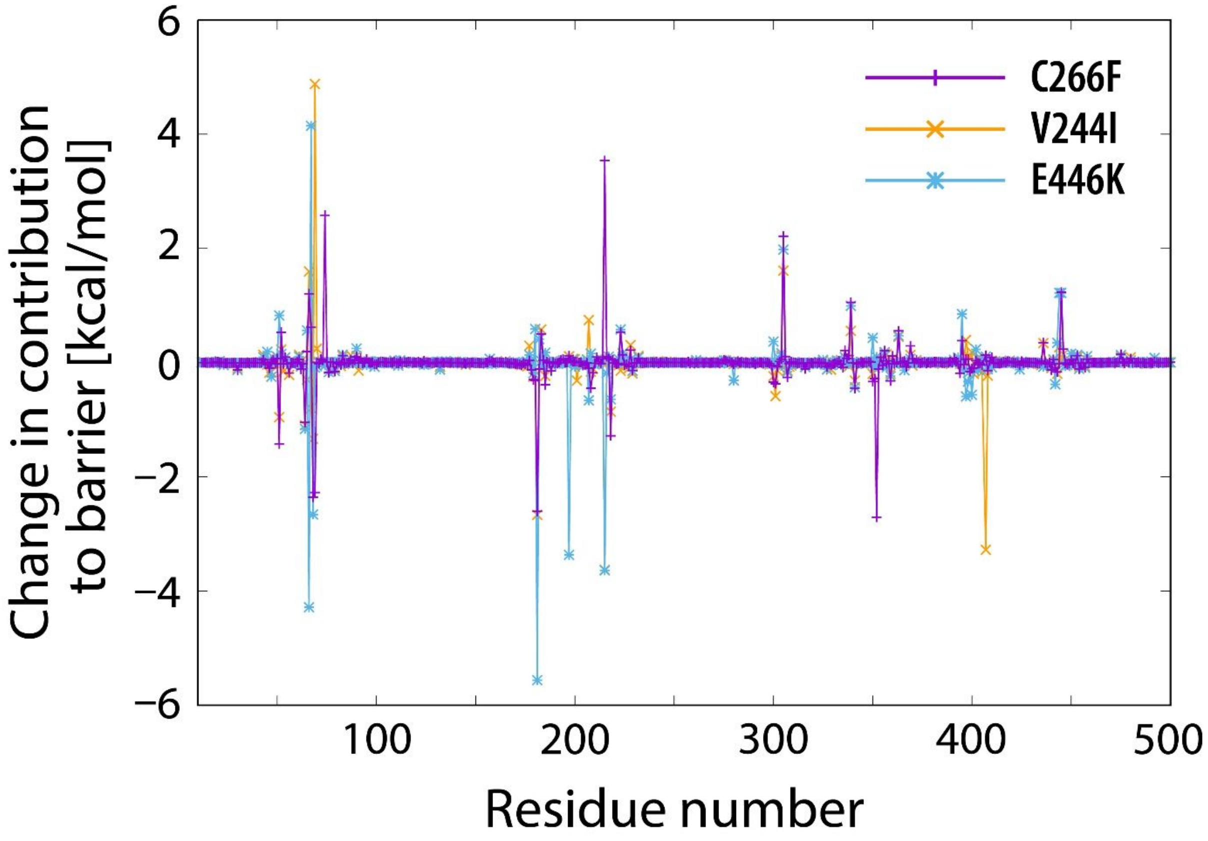

Figure S3. Differential plots of contributions of individual residues of three MAO-A mutants (C266F, V244I and E446K) to the catalytic performance of the enzyme, relative to WT MAO-A. Negative values indicate that the corresponding residues act more catalytically in the mutant than in WT MAO-A, and *vice versa* for the positive values. The contributions of individual residues have been computed for the mutants in the same way as for the WT enzyme (see Fig. S2 and Sections 2 and 3), including altogether 307,200 single point calculations using PM6 semiempirical quantum chemical approach. As mentioned for WT MAO-A (Fig. S2), the employed PM6 method is of limited accuracy, and the values of individual points are disputable. However, the observed trends (few and narrow catalytically active domains, less than 10% of residues contributing to the net effect of point mutation, etc.) appear to be valid. Note the similarity of all three patterns to the one displayed in Fig. S2.
